# No time to die: Comparative study on preservation protocols for anaerobic fungi

**DOI:** 10.3389/fmicb.2022.978028

**Published:** 2022-09-26

**Authors:** Julia Vinzelj, Akshay Joshi, Diana Young, Ljubica Begovic, Nico Peer, Lona Mosberger, Katharina Cécile Schmid Luedi, Heribert Insam, Veronika Flad, Magdalena Nagler, Sabine Marie Podmirseg

**Affiliations:** ^1^Department of Microbiology, University of Innsbruck, Innsbruck, Austria; ^2^Institute of Chemistry and Biotechnology, Biocatalysis and Process Technology Unit, Zurich University of Applied Sciences, Wäedenswil, Switzerland; ^3^Micro- and Molecular Biology, Central Department for Quality Assurance and Analytics, Bavarian State Research Center for Agriculture, Freising, Germany

**Keywords:** Neocallimastigomycota, cryopreservation, anaerobic fungi, long-term storage, short-term storage, preservation techniques, resting stages, culture preservation

## Abstract

Anaerobic fungi (AF, phylum Neocallimastigomycota) are best known for their ability to anaerobically degrade recalcitrant lignocellulosic biomass through mechanic and enzymatic means. While their biotechnological potential is well-recognized, applied research on AF is still hampered by the time-consuming and cost-intensive laboratory routines required to isolate, maintain, and preserve AF cultures. Reliable long-term preservation of specific AF strains would aid basic as well as applied research, but commonly used laboratory protocols for AF preservation can show erratic survival rates and usually exhibit only moderate resuscitation success for up to one or two years after preservation. To address both, the variability, and the preservation issues, we have set up a cross-laboratory, year-long study. We tested five different protocols for the preservation of AF. The experiments were performed at three different laboratories (Austria, Germany, Switzerland) with the same three morphologically distinct AF isolates (*Anaeromyces mucronatus, Caeocmyces sp.*, and *Neocallimastix cameroonii*) living in stable co-culture with their naturally occurring, syntrophic methanogens. We could show that handling greatly contributes to the variability of results, especially in *Anaeromyces mucronatus*. Cryopreservation of (mature) biomass in liquid nitrogen had the highest overall survival rates (85–100%, depending on the strain and laboratory). Additionally, preservation on agar at 39°C had surprisingly high survival rates for up to 9 months, if pieces of agar containing mature AF thalli were resuscitated. This low-cost, low-effort method could replace consecutive batch cultivation for periods of up to 6 months, while long-term preservation is best done by cryopreservation in liquid nitrogen. Regardless of the method, however, preserving several replicates (>three) of the same strain is highly advisable.

## Introduction

Anaerobic fungi (AF, phylum Neocallimastigomycota) belong to the basal lineages of fungi and are mainly known for their uncanny ability to break down lignocellulosic material under anaerobic conditions. To date, 20 genera of Neocallimastigomycota have been described (Hess et al., 2020; Stabel et al., 2020; Hanafy et al., 2021), and several more are expected from cultivation-independent studies (Paul et al., 2018; Hanafy et al., 2020). In addition to their morphological variability, AF genera and species can exhibit differences in enzymatic activities (Paul et al., 2010; Henske et al., 2018) and general metabolism (Griffith et al., 2009). While the biotechnological potential of AF is now widely recognized (Flad et al., 2020; Swift et al., 2021), scientists are still struggling with the basics of culture handling, and the time-consuming culture care is making AF cultivation expensive and arduous. AF strains are usually kept in consecutive batch culture that requires routine sub-cultivation every 3–14 days (depending on the medium and medium volume they are kept in). Cultivation techniques and media recipes have evolved over time (Orpin, 1975; Zhu et al., 1996; Theodorou et al., 2005; Peng et al., 2018; Stabel et al., 2021), but there is still no standardized medium and each research group is using a slightly different cultivation technique. Furthermore, despite best efforts, several strains that were isolated before the year 2000 were lost over time (Hanafy et al., 2022) due to the laborious maintenance routine and the complex, unknown nutrient requirements of AF. Research dedicated to long-term preservation techniques for AF is sparse. Early publications reported cryopreservation in liquid nitrogen with 5% v/v dimethyl sulfoxide (DMSO) as cryoprotectant (Yarlett et al., 1986), but survival rates after one year of preservation were as low as 40%. Supplementation of the cultivation and preservation medium with 0.5% w/v agar and freezing at –80°C (Kostyukovsky et al., 1995) kept AF viable for up to 4 months. Using ethylene glycol instead of DMSO and adding sterile rumen fluid, brought survival rates up to 80% after one year (Sakurada et al., 1995). Nagpal et al. (2012) showed that *Caecomyces* was best maintained in glycerol at –70°C and remained viable for 3 months, and Solomon et al. (2016) could show that preservation in 15% glycerol at –80°C kept some of the tested AF cultures alive for up to two years. Besides cryopreservation, preservation on agar has been reported successful for up to seven months (Joblin, 1981, Calkins et al., 2016). All those publications, however, reported different methods for preservation, freezing, resuscitation, and cultivation. Additionally, all methods were performed on different AF genera and strains, and the reporting of methods and results were sometimes incomplete. Hence, no clear standard method for preservation of AF could be derived from those publications. Due to the metabolic variability between AF strains and the biotechnological interest in AF, reliable, low-effort preservation techniques are urgently needed for basic as well as applied research. Developing a standardized and reliable preservation technique for AF could also enable their deposition in general culture collections which would ultimately enable more research on AF and their biotechnological applications.

**FIGURE 1:**
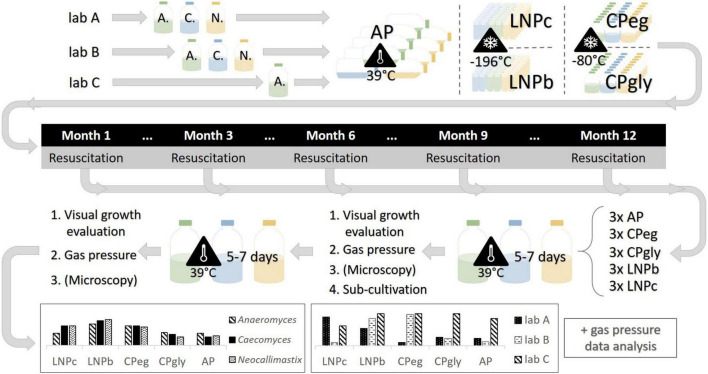
Experimental design of this study. *A*., *Anaeromyces mucronatus*; C., *Caecomyces* sp.; N., *Neocallimastix cameroonii*. AP, agar preservation protocol; CPeg, cryopreservation protocol with ethylene glycol stock solution; CPgly, cryopreservation protocol with glycerol stock solution; LNPb, preservation of liquid culture with ethylene glycol stock solution in liquid nitrogen; LNPc, preservation of liquid culture with ethylene glycol stock solution in liquid nitrogen.

In this study, we aimed to shed light on the preservation of AF by testing five different preservation protocols on three different AF strains. Those strains were growing in stable co-culture with their naturally occurring, syntrophic methanogens and were chosen based on AF morphology. Syntrophic AF-methanogen co-cultures are the predominant product in AF isolation if one does not actively inhibit the growth of methanogens (e.g., by the addition of chloramphenicol or 2-bromoethanesulfonate). Furthermore, co-cultures of AF and methanogens might be of higher interest for biotechnology since they have been shown to be enzymatically more active than pure cultures (Swift et al., 2019; Li et al., 2020; Ma et al., 2020). For this study, *Anaeromyces mucronatus* served as a representative of filamentous, polycentric AF, *Neocallimastix cameroonii* as a representative of filamentous, monocentric AF, and *Caecomyces sp.*, as representative of bulbous, monocentric AF. To further detangle true effects of the different preservation protocols from differences in culture handling, the same experiments were performed in two different laboratories (lab A and lab B) with the same three strains. Additionally, the same experiments were also performed on the same *Anaeromyces mucronatus* strain in a third lab (lab C) that had additional expertise in handling this culture. To evaluate the protocols for their preservation efficacy over time, batches of preserved cultures were resuscitated after one week, three months, six months, nine months, and one year of preservation (see Figure 1). The overall goal of this study was to define the preservation technique best suited for each morphological group of AF or, if possible, to define a generally valid protocol for the preservation of AF.

## Materials and methods

### Strains

The strains used for this experiment were growing in stable co-cultures with the naturally occurring prokaryotes they were isolated with (see Table 1). The co-cultures were shared between all three partnering laboratories by transporting growing cultures in serum bottles within portable incubators at 39°C by train or car (transporting time <4 h). The AF in each culture were identified via sequencing using the previously described, AF specific GGNL primer pair (Nagler et al., 2018). Syntrophic prokaryotes were identified by PCR and Sanger sequencing of the V4 region of 16S rRNA, using the primer pair 515f and 806r (Caporaso et al., 2011). Strain YoDo11 is a co-culture of *Neocallimastix cameroonii* and more than one prokaryote. The presence of at least one methanogen in this co-culture has been verified by fluorescence microscopy (F_420_ autofluorescence) and PCR with primers targeting the *mcrA* gene (Angel et al., 2011). During the experiment, microscopy was used to verify the presence of methanogens. Prior to experiments, all co-cultures were continuously cultivated in serum bottles (with a total volume of 120 mL) containing 50 mL of medium A (described below) lacking cellobiose, and sub-cultured weekly (at lab A and lab B) or bi-weekly (at lab C). The cultures were incubated stationary in the dark at 39°C. Incubation times right before preservation are specified below for each tested protocol and were the same for every institute.

**TABLE 1:** Strains used in this study.

Strain name	ViSuPo	YoDo3	YoDo11
Anaerobic fungi	*Caecomyces* sp.	*Anaeromyces mucronatus*	*Neocallimastix cameroonii*
Syntrophic prokaryotes	*Methanobrevibacter sp.*	*Methanobrevibacter sp.*	Unidentified mixture of prokaryotes; at least one methanogen
Isolation year	2019	2017	2017
Isolation source	feces	feces	feces
Host organism	*Rupicapra rupicapra*	*Llama llama*	*Giraffa camelo-pardalis reticulata*
Sequence Accession Number	AF: OP216660 methanogen: OP176002	AF: ON614226 to ON614231 methanogen: OP175998 to OP175999	AF: ON614553 to ON614554, ON614569 to ON614571, ON614594, ON614595 prokaryotes: at least one methanogen; not conclusively identified yet

All strains used in this study represent co-cultures of anaerobic fungi (AF, phylum Neocallimastigomycota) and prokaryotes. AF have been identified by sequencing of the D1/D2 region of the LSU (primers GGNL1F and GGNL4R). The presence of methanogens was verified by fluorescence microscopy, PCR (against the mcrA gene, primers mlas-mod-f and mcrA-rev), and/or sequencing of the V4 region (primers 515f and 806r).

### Clarified rumen fluid

Clarified rumen fluid (CRF) was provided by lab B for all other partnering labs (shipped sterile and frozen). For preparation of CRF, fresh cattle rumen content was obtained from a slaughterhouse. The rumen content was first sieved, then passed through two layers of cheese cloth, autoclaved (121°C, 20 min) and then centrifuged at 10,000 × *g* for 15 min. The aliquoted supernatant was used for medium preparation. It was stored at –20°C until use.

### Stock solutions

For this experiment series several stock solutions were prepared. Salt solution I was prepared by dissolving 3 g K_2_HPO_4_ in 1 L MilliQ water. Salt Solution II consisted of 3 g/L KH_2_PO_4_, 6 g/L (NH_4_)_2_SO_4_, 6 g/L NaCl, 0.6 g/L MgSO_4_.7H_2_0, 0.6 g/L CaCl_2_.2H_2_O dissolved in MilliQ water. Both solutions were autoclaved (121°C, 20 min) and stored at 4°C until use. Hemin stock solution consisted of 0.05% w/v hemin powder in a 1:1 mixture of 96% ethanol and 0.05 M NaOH solution. It was filter sterilized (0.22 μm pore size) and stored at 4°C. To suppress growth of bacteria in the AF-methanogen co-cultures antibiotic stock solution was used. It consisted of 5 mg/mL of each Streptomycin sulfate, Penicillin G sodium salt, and Ampicillin sodium salt dissolved in water. It was filter sterilized (0.22 μm pore size), stored at 4°C, and added to the culture bottles by injection shortly before use (0.5 mL per 50 mL medium). The growth of methanogens was not intentionally inhibited before or at any point during the experiment. Ethylene glycol stock solution was prepared by mixing 49.7 g of ethylene glycol (p.a. > 99.5%) with 155 mL of CRF, 0.2 mL resazurin stock solution, 0.2 g L-cysteine-HCl, and 1.2 g NaHCO_3_. The mixture was gassed with pure CO_2_ for 30 min, then aliquoted into serum bottles (pre-gassed with CO_2_), closed with a butyl rubber stopper, crimped, autoclaved (121°C, 20 min), and stored at 4°C until use. For the glycerol stock solution, a 60% glycerol solution was aliquoted into serum bottles and gassed with CO_2_ for 30 min. The bottles were then closed with a butyl rubber stopper, crimped, autoclaved (121°C, 20 min), and stored at 4°C until use.

### Media preparation

For one liter of cultivation medium, 150 mL salt solution I and 150 mL salt solution II were mixed with 3 g yeast extract, 10 g tryptone, 2 mL hemin stock solution, 2 mL resazurin stock solution, 2 g xylan powder (from beechwood), and 850 mL distilled or MilliQ water. The mixture was heated until the liquid changed color and then cooled on ice while gassing it with pure CO_2_. Once cooled to room temperature, 150 mL CRF was added together with 6 g NaHCO_3_, 3 g cellobiose, and 1 g L-cysteine under constant CO_2_. Once the liquid exhibited a brown-yellowish color, pH was set to 6.9 with the help of 5 M NaOH solution. The medium was then aliquoted into the appropriate pre-gassed serum bottles which were then closed with a butyl rubber stopper, and crimped After autoclaving (121°C, 20 min), the bottles were stored in darkness either at 37°C (lab B) or room temperature (lab C, lab A) until use.

In this experiment, three different serum bottles with N20 crimp tops were used: “large” serum bottles with a total volume of 120 mL, “small” serum bottles with a total volume of 60 mL, and serum bottles with a total volume of 16 mL (hereafter referred to as “glass vials”). If not otherwise specified, transfer of cultures between serum bottles was done by injection. 18G cannulas (1.20 mm × 40 mm) and 5 mL syringes were used for culture transfer, and 20G cannulas (0.90 mm × 40 mm) were used for transfer of medium B. Injection of antibiotic stock solution was performed with 24G needles (0.55 mm × 25 mm) and 1 mL syringes.

For medium A, 5 mg/mL of dried, and milled wheat straw (size <2 mm) were added to the serum bottles before gassing them with pure CO_2_ and dispensing 50 mL medium into the large serum bottles and 30 mL into the small ones. In medium B, no wheat straw was added. Agar bottles for protocol AP (see below) contained medium B and were prepared by adding 2% w/v agar (Kobe I) to large serum bottles before they were gassed and the medium (10 mL per bottle) was dispensed. After autoclaving, agar bottles were stored together with the other bottles until use.

### Preservation protocols

At lab A and lab B, all protocols were performed with all strains. Additionally, lab C tested all protocols with *Anaeromyces mucronatus* only. Prior to preservation, AF cultures were incubated stationary in the dark at 39°C for 3–5 days. Only cultures with good growth and sufficient biomass were selected for preservation. Per protocol and strain at least 15 replicates were preserved at once to enable resuscitation of three replicates per strain and protocol at each resuscitation timepoint.

#### Preservation on agar (AP, preservative: none)

**FIGURE 2:**
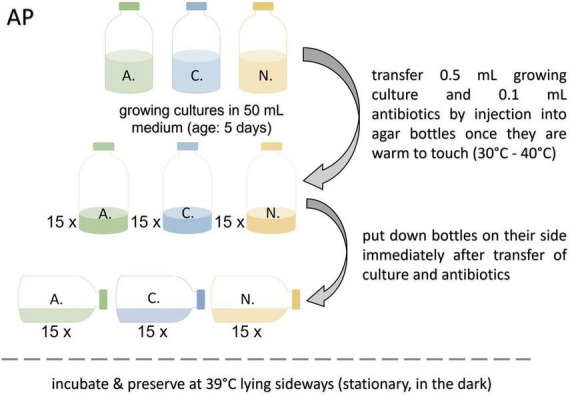
Workflow for the agar preservation protocol (AP). A., *Anaeromyces mucronatus*; C., *Caecomyces* sp.; *N.*, *Neocallimastix cameroonii*.

The agar bottles prepared for this protocol were heated to 80°C shortly before use to melt the agar. After cooling down to approx. 30–40°C (warm to touch) 0.1 mL of antibiotic stock solution, and 0.5 mL of growing culture (not older than 5 days) were added. The bottles, laying on their sides, were incubated in darkness at 39°C until resuscitation (see Figure 2). This protocol was based on the method for spore extraction reported by Calkins et al. (2016).

#### Cryopreservation at –80°C in ethylene glycol stock solution (CPeg)

**FIGURE 3:**
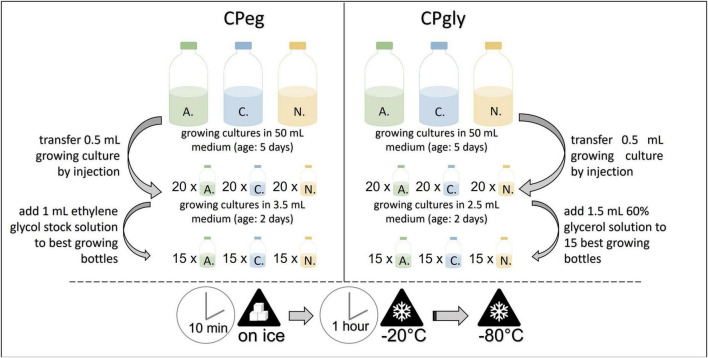
Workflow for the cryopreservation protocols CPeg **(left)** and CPgly **(right)**. *A.*, *Anaeromyces mucronatus*; C., *Caecomyces* sp.; N., *Neocallimastix cameroonii.* CPeg, cryopreservation protocol with ethylene glycol stock solution; CPgly, cryopreservation protocol with glycerol stock solution.

Small glass vials, each filled with 3.5 mL of medium A, were inoculated by injecting 0.5 mL of the same, growing culture. After incubation at 39°C for two days, 15 glass vials with the best growth were chosen per strain and injected with 1 mL pre-cooled ethylene glycol stock solution each. The glass vials were put on ice for 10 min and then transferred to –20°C for 1 h. They were stored at –80°C until resuscitation (see Figure 3). This protocol was based in part on the method reported by Nagpal et al. (2012).

#### Cryopreservation at –80°C in glycerol stock solution (CPgly)

Small glass vials, each filled with 2.5 mL of medium A, were inoculated by injecting 0.5 mL of the same, growing culture. After incubation at 39°C for two days, 15 glass vials with the best growth were chosen per strain and injected with 1.5 mL pre-cooled glycerol stock solution each. The glass vials were put on ice for 10 min and then transferred to –20°C for 1 h. They were stored at -80°C until resuscitation (see Figure 3). This method was based in part on the method reported by Nagpal et al. (2012).

#### Preservation of biomass clumps in liquid nitrogen (LNPb, preservative: Ethylene glycol stock solution)

**FIGURE 4:**
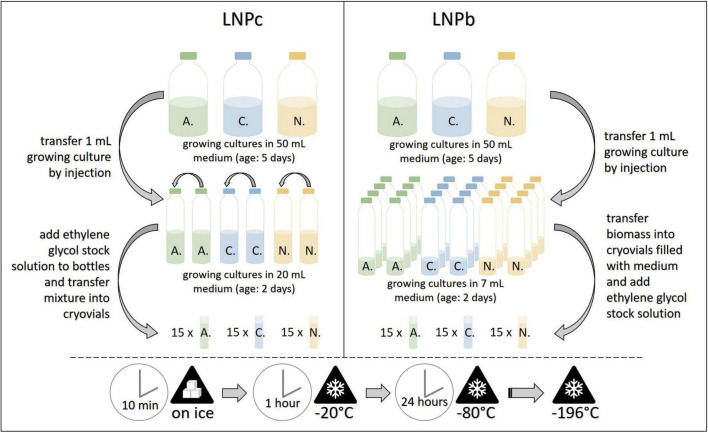
Workflow for the cryopreservation protocols LNPc **(left)** and LNPb **(right)**. A., *Anaeromyces mucronatus*; C., *Caecomyces* sp.; N., *Neocallimastix cameroonii.* –196°C refers to preservation in liquid nitrogen. LNPb, preservation of liquid culture with ethylene glycol stock solution in liquid nitrogen; LNPc, preservation of liquid culture with ethylene glycol stock solution in liquid nitrogen.

For this protocol several small serum bottles filled with 7 mL medium A (approx. one per two cryovials per strain) were prepared. The small serum bottles were inoculated by injection with 1 mL of growing culture and incubated at 39°C for 2 days. Then, plastic cryovials (ThermoScientific, Art.No. 377267) were opened in a sterile environment (laminar flow cabinet) and each filled with 1 mL sterile medium B. For filamentous AF strains, the serum bottles were opened, and fungal biomass clumps or mats were transferred with sterilized tweezers into the cryovials. For the bulbous AF, the serum bottles were opened and the whole content transferred into sterile 50 mL plastic tubes. After centrifugation (4,000 × *g*, 1 min), most of the supernatant was discarded, and with the help of a pipette and cut tips, 1 mL of the remaining fungi-straw suspension was transferred into the cryovials. After culture transfer, 1 mL of pre-cooled ethylene glycol stock solution was added to all cryovials. The cryovials were then closed, inverted, and put on ice for 10 min, frozen at –20°C for 1 h and put on –80°C for 24 h. They were then transferred into liquid nitrogen, where they were stored in the gaseous phase (lab A) or liquid phase (lab B, lab C) until resuscitation (see Figure 4). This protocol was based on the method reported by Callaghan et al. (2015) and Solomon et al. (2016).

#### Preservation of cultures in liquid nitrogen (LNPc, preservative: Ethylene glycol stock solution)

For this protocol, two small serum bottles filled with 20 mL medium A were prepared and inoculated by injection with 1 mL growing culture each. After incubation at 39°C for two days, these starter cultures were unified by transferring 5 mL of one bottle into the other. This bottle was then injected with 6.25 mL of pre-cooled ethylene glycol stock solution, mixed well, and put on ice for five minutes. Then, 2 mL of the mixture were transferred into sterile plastic cryovials with the help of needle and syringe. The subsequent freezing procedure with the filled cryovials followed that of protocol LNPb (see Figure 4). At each institute, 15 cryovials per strain were preserved. This protocol was based on the method reported by Callaghan et al. (2015).

### Resuscitation protocols

Resuscitation of three replicates per strain and protocol was performed at five distinct time points: one week, three months, six months, nine months, and 12 months after preservation. Resuscitation was performed either in small serum bottles (total volume of 60 mL, filled with 30 mL medium A), and/or large serum bottles (total volume of 120 mL, filled with 50 mL medium A) as specified below. Antibiotic stock solution (0.1 mL per 10 mL medium) was added to the serum bottles shortly before resuscitation. After 5–7 days of stationary incubation at 39°C in the dark, AF growth in the resuscitated bottles was evaluated (see below) and, if growth could be observed, one resuscitation bottle (large or small) per strain, replicate, and protocol was sub-cultivated by injection (5 mL) into a fresh large serum bottle. Antibiotic stock solution (0.1 mL per 10 mL medium) was also added to these fresh serum bottles shortly before inoculation. After incubation for 5–7 days, growth of this second bottle was evaluated and the bottle afterward discarded.

#### Resuscitation for the AP protocol

For resuscitation of the cultures preserved on agar, the agar bottles were taken out of storage and 10 mL sterile medium B were carefully injected, not disturbing the surface of the agar too much. After incubation for one hour at 39°C (lying sideways), the injected medium was taken out again and transferred into a large serum bottle filled with sterile medium A. This large serum bottle was incubated at 39°C (stationary, in the dark) for five to seven days. If growth could be observed, this bottle was sub-cultivated as described above.

#### Resuscitation for protocols CPeg and CPgly

For resuscitation of the cultures preserved at –80°C, the glass vials were taken out of storage and thawed in a 39°C water bath for 5 min. After shaking it gently, 2 mL were taken out of the vial and transferred into a large serum bottle filled with sterile medium A. Then, 5 mL of sterile medium A were injected into each glass vial and both, the large serum bottles and the glass vials were incubated at 39°C (stationary, in the dark) for five to seven days. Depending on growth evaluation, either the glass vial or the large serum bottle was sub-cultivated.

#### Resuscitation for protocols LNPb and LNPc

For resuscitation of the cultures preserved in liquid nitrogen, the cryovial was taken out of storage and thawed in a 39°C water bath for 5 min. In a sterile environment (laminar flow cabinet), a small serum bottle filled with sterile medium A was opened and the content of the cryovial poured in. The bottle was immediately re-sealed and shaken well. Then, 5 mL were transferred from the small serum bottle into a large serum bottle filled with sterile medium A by injection (to dilute the preservative). Both bottles, small and large, were incubated at 39°C (stationary, in the dark) for 5–7 days. Depending on growth evaluation, either the small or the large serum bottle was sub-cultivated.

### Growth evaluation

Gas pressure measurements (Theodorou et al., 1994) as well as visual inspection were used for growth evaluation. Per strain, two to three bottles filled with 50 mL medium A and injected with 5 mL growing culture were used as a positive control. Additionally, one to three serum bottles filled with 50 mL medium A were injected with 5 mL sterile medium and were used as negative controls. Positive and negative controls were prepared and incubated together with the resuscitated AF cultures. Before incubation, gas was released from all bottles to level the pressure. Accumulated gas pressure after incubation was measured either at 37°C (at lab B), or one by one immediately after taking the bottles out of the incubator (lab A and lab C), to ensure correct measurements. At lab A, the compact LEO2 digital manometer from Keller Druckmesstechnik AG was used for gas pressure measurements. At lab B, the GDH 200-13 and at lab C the GMH 3111 from GHM GROUP Greisinger was used.

Successful growth was visually detected by formation of mats/buoys or biomass spheres/balls in filamentous AF (*Neocallimastix* and *Anaeromyces*), or suspended biomass flakes for bulbous AF (*Caecomyces*). Visual inspection also evaluated the redness (indicating less anoxic conditions) and turbidity (indicating bacterial contamination) of the medium. Visible growth as well as redness and turbidity of the medium was rated on a scale of “-”(none), “∼,” “+,” “++,” to “+++” (high). Unification of evaluation between the institutes was attempted by a catalog of reference pictures (Supplementary Figures 1–4). If growth of AF was not immediately obvious (lack of fungal biomass), microscopy was used to determine the presence of fungal structures associated with well-growing cultures. This included the presence/absence of zoospores, attachment of vegetative AF to straw particles, and the overall number of complete fungal thalli found.

### Data filtering and statistical analysis

Statistical analysis of the gas pressure data was done by analysis of patterns between the continuous variable accumulated gas pressure (in bar) and categorical variables like strain, institute, and growth. Only valid gas pressure data (no leaky stoppers, no loss of gas pressure during measurements) were used in the analysis. Gas pressure data from bottles with bacterial contamination was also excluded from downstream analysis. Statistical analysis and visualizations were done in R (version 4.2), using the packages tidyverse (version 1.3.1; Wickham et al., 2019), ggpubr (version 0.4.0, Kassambara, 2020), rstatix (version 0.7.0, Kassambara, 2021), broom (version 0.7.10, Robinson et al., 2022), and ggStatsplot (version 0.9.1, Patil, 2021). The seed was set to 042022. For each analysis, outliers were first removed using the identify outliers function of the rstatix package. Then compliance with the requirements for ANOVA (normally distributed residuals, homogeneity of variance) was tested using the shapiro_test and levene_test functions of the rstatix package. If one of those requirements was not met, non-parametric tests were performed. For comparisons between two groups, Mann–Whitney *U* tests were performed. For comparisons between more than two groups, a Kruskal-Wallis test with *post hoc* Dunn test analysis was performed. Those tests were also performed with the respective functions found in the rstatix package. Visualization including statistical data analysis was done with the function ggbetweenstats found in the ggStatsplot package.

## Results

### Results by protocol

Considering the data from all institutes, all strains, and all timepoints, 63.8% of attempted AF resuscitations were successful for protocol LNPb, while CPeg had a success rate of 52.4%. The overall success rates for protocols LNPc, CPgly, and AP were below 50% (see Table 2). The prokaryotic partners of all strains survived preservation and resuscitation as long as the AF did too.

**TABLE 2:** Overall survival rates.

Strain	AP (%)	CPeg (%)	CPgly (%)	LNPb (%)	LNPc (%)	CP (%)	LN (%)	EG (%)
**Successful resuscitations summarized over all institutes, broken down per protocol and strain**								
*Anaeromyces*	33.3	53.3	35.6	57.8	33.3	44.4	45.6	48.1
*Caecomyces*	23.3	53.3	30.0	66.7	53.3	41.7	60.0	57.8
*Neocallimastix*	10.0	50.0	23.3	70.0	53.3	36.7	61.7	57.8
Successful total	23.8	52.4	30.5	63.8	44.8	41.4	54.3	53.7
**Successful resuscitations summarized over all strains, broken down per protocol and institute**								
**Institute**								
Lab A	20.0	8.9	22.2	46.7	77.8	11.7	46.7	33.3
Lab B	11.1	84.4	20.0	73.3	8.9	52.2	41.1	55.6
Lab C	73.3	86.7	86.7	86.7	53.3	86.7	70.0	75.6
Successful total	23.8	52.4	30.5	63.8	44.8	41.4	54.3	53.7

Successful resuscitations are given as percentage of attempted resuscitations. AP, agar preservation protocol; CPeg, cryopreservation protocol with ethylene glycol stock solution; CPgly, cryopreservation protocol with glycerol stock solution; LNPb, preservation of biomass with ethylene glycol stock solution in liquid nitrogen; LNPc, preservation of liquid culture with ethylene glycol stock solution in liquid nitrogen; CP, combined survival rates of CPeg and CPgly; LN, combined survival rates of LNPb and LNPc; EG, combined survival rates of CPeg, LNPb, and LNPc.

#### Results for protocol AP

In general, protocol AP performed worst out of all protocols. Viable spores and/or scattered AF thalli from the agars’ surface could be extracted by flushing after one week of preservation to some degree (see Supplementary Table 1). After that timepoint, flushing the agar bottles had only poor success (below 0.2%) at all labs. At lab C, however, two methods of breaking the agar have been successfully used after flushing attempts failed. After one week, three months, and six months of preservation, the agar bottles were roughly shaken after injection with medium B (similar to what was reported in Calkins et al., 2016) to extract some mature AF thalli from deeper layers of the agar plate. This led to a resuscitation success of 100% after one week and after three months, but at the six-month mark only one out of three replicates could be revived that way. At the nine-month and 12-month mark of preservation, this method again did not work. Therefore, the bottles were opened and small agar pieces containing mature AF thalli were transferred into large serum bottles containing 50 mL sterile, anoxic medium A. This led to one survivor out of three at the nine-month mark, and two out of three at the 12-month mark. At lab B, this method was also attempted after nine and 12 months of preservation. It led to high survival rates (three out of three for *Anaeromyces* and *Neocallimastix*, two out of three for *Caecomyces*) after nine months, but only one successful resuscitation for *Caecomyces* (and none for the others) after 12 months. At lab A these additional steps were not attempted.

#### Results for protocol CPeg

Protocol CPeg had high overall success rates at lab B and lab C (84.4, and 86.7%, respectively), but low performance rates at lab A (see Table 3). At lab B, CPeg worked best for *Neocallimastix* (100% of all replicates over all timepoints were successfully revived), followed by *Caecomyces* (86.7%), and *Anaeromyces* (66.7%). At lab C, all replicates of *Anaeromyces* survived up until the nine-month mark, while only one replicate survived after 12 months. At lab A, CPeg showed only some success one week after preservation, but none beyond that time.

**TABLE 3:** Survival rates per strain in each lab.

Strain	AP (%)	CPeg (%)	CPgly (%)	LNPb (%)	LNPc (%)	CP (%)	LN (%)	EG (%)
**Lab A: Successful resuscitations, broken down per protocol and strain**								
*Anaeromyces*	20.0	6.7	20.0	0.0	46.7	13.3	23.3	17.8
*Caecomyces*	33.3	20.0	46.7	100.0	93.3	33.3	96.7	71.1
*Neocallimastix*	6.7	0.0	0.0	40.0	93.3	0.0	66.7	44.4
Successful total	20.0	8.9	22.2	46.7	77.8	15.6	62.2	44.4
**Lab B: Successful resuscitations, broken down per protocol and strain**								
*Anaeromyces*	6.7	66.7	0.0	86.7	0.0	33.3	43.3	51.1
*Caecomyces*	13.3	86.7	13.3	33.3	13.3	50.0	23.3	44.4
*Neocallimastix*	13.3	100.0	46.7	100.0	13.3	73.3	56.7	71.1
Successful total	11.1	84.4	20.0	73.3	8.9	52.2	41.1	55.6
**Lab C: Successful resuscitations, broken down per protocol and strain**								
*Anaeromyces*	73.3	86.7	86.7	86.7	53.3	86.7	70.0	75.6

Successful resuscitations are given as percentage of attempted resuscitations. AP, agar preservation protocol; CPeg, cryopreservation protocol with ethylene glycol stock solution; CPgly, cryopreservation protocol with glycerol stock solution; LNPb, preservation of biomass with ethylene glycol stock solution in liquid nitrogen; LNPc, preservation of liquid culture with ethylene glycol stock solution in liquid nitrogen; CP, combined survival rates of CPeg and CPgly; LN, combined survival rates of LNPb and LNPc; EG, combined survival rates of CPeg, LNPb, and LNPc.

#### Results for protocol CPgly

Protocol CPgly had an overall success rate of 30.5%. Like protocol CPeg, it showed high survival rates at Lab C (*Anaeromyces*) up until the nine-month mark, but after that only one replicate was successfully revived. This high success rate, however, did depend heavily on additional handling steps that were only performed at lab C. Those steps included the opening of bottles and transfer of biomass pieces with tweezers whenever sub-cultivation by injection did not work. Without those modifications, CPgly did not lead to viable cultures after resuscitation at lab C (Supplementary Table 1). For lab A and lab B, CPgly showed little to no success over time. At lab A, it showed an overall success rate of 46.7% for *Caecomyces*, but *Anaeromyces* could only be revived after one week of preservation, while *Neocallimastix* did not survive at all. At lab B, protocol CPgly had a success rate of 46.7% for *Neocallimastix*, but only 13.3% for *Caecomyces*, and *Anaeromyces* did not survive at all.

#### Results for protocol LNPb

Protocol LNPb had relatively high success rates at all labs. At lab C (*Anaeromyces*) 13 out of all 15 replicates were successfully resuscitated (86.7% success rate). At lab B, it worked best for *Neocallimastix* (100%) and *Anaeromyces* (86.7%), but not as well for *Caecomyces* (33.3%). Interestingly, at lab A LNPb worked best for *Caecomyces* (100%), but not well for *Anaeromyces* and *Neocallimastix* (0, and 40%, respectively).

#### Results for protocol LNPc

For lab A, LNPc worked best out of all protocols, with 49.7, 93.3, and 93.3% of attempted resuscitations ending in success for *Anaeromyces*, *Caecomyces*, and *Neocallimastix*, respectively. For lab B, protocol LNPc had the lowest success rate out of all protocols with only 4 out of 45 replicates (all strains and all timepoints combined) successfully resuscitated (8.9% of attempted LNPc resuscitations at lab B). At Lab C, LNPc showed moderate success rate with 53.3% of *Anaeromyces* replicates being resuscitated successfully.

### Survival after one year of preservation

After one year of preservation, three out of three replicates of LNPb for *Anaeromyces* survived each at lab B and Lab C. Additionally, three out of three replicates of CPeg for *Anaeromyces* survived at lab B, while two out of three replicates of each, LNPc and AP, were resuscitated at Lab C. At lab A none of the replicates of *Anaeromyces* survived. For *Caecomyces*, three out of three replicates of LNPc and LNPb survived at lab A. At lab B, however, survival rates for this strain were very low, with only one replicate surviving each for protocol CPeg, CPgly, and LNPc. Conversely, success rates for *Neocallimastix* were low at lab A (one out of three replicates for LNPb, two out of three for LNPc, none for all other protocols), while three out of three replicates of each CPeg, CPgly, and LNPb survived at lab B.

### Results by institute

The overall survival rate *for Anaeromyces* replicates was 76.0% at lab C. This high success rate was mostly due to the aforementioned additional handling steps performed at lab C. Without those, survival rate for *Anaeromyces* was 44.0% at lab C. These extra handling steps were most influential in protocols AP, and CPgly (Supplementary Table 1). Lab A showed most success in handling *Caecomyces* (58.7% overall success rate), and lower success rates with *Neocallimastix* (28.0%) and *Anaeromyces* (18.7%). At lab B, 54.7% of the preserved replicates of *Neocallimastix* were revived, while only 32.0% of both, *Anaeromyces* and *Caecomyces*, could be successfully resuscitated.

For lab A, LNPc showed highest success (77.8%). All other protocols had a success rate of below 50%. At lab B, CPeg and LNPb worked best in general, with success rates of 84.4 and 73.3%, respectively. LNPc, CPgly, and AP performed worst with success rates below 50%. At Lab C, where the focus was on *Anaeromyces* only, protocol CPeg, CPgly, and LNPb showed equally high success rates with 86.7%. Protocols AP and LNPc at lab C showed success rates of 66.7 and 53.3%, respectively.

### Results by strain

#### Anaeromyces

At lab A, overall success with *Anaeromyces* was low. LNPc worked best with a survival rate of 46.7%. At the 12-month mark, no replicates of *Anaeromyces* survived at lab A. At lab B, LNPb (86.7%) and CPeg (66.7%) were the only protocols working for *Anaeromyces*. At the 12-month mark, all replicates of those two protocols survived. At Lab C, protocols CPeg, CPgly, and LNPb showed the highest overall success rates for *Anaeromyces*. However, CPeg and CPgly showed a significant drop in survival success at the 12-month mark with only one replicate each surviving, while LNPb showed undisturbed resuscitation success.

At lab A, liquid nitrogen preservation had a higher success rate than cryopreservation for *Anaeromyces* (23.3 vs. 13.3%). The same was true for lab B (43.3% vs. 33.3%). At lab C, however, cryopreservation (86.7%) performed slightly better than liquid nitrogen preservation (70.0%), because LNPc was the only protocol with comparatively low success rate (53.3%).

#### Caecomyces

At lab A, *Caecomyces* showed highest overall survival rates with LNPb (100%) and LNPc (93.3%). At the 12-month mark all replicates of those two protocols were successfully resuscitated. CPgly (46.7%) worked slightly better than CPeg (20.0%), but both protocols had poor outcomes compared to LNPc and LNPb. At lab B, CPeg worked best for *Caecomyces* with a survival rate of 86.7%. Up until nine months of preservation, all replicates of CPeg survived. At the 12-month mark, only one replicate was successfully revived. LNPb showed mixed results for lab B, with all replicates surviving at the nine-month mark, but none surviving at the three-, six-, and 12-month marks. LNPc and CPgly showed poor, mixed results (see Supplementary Table 1). At the 12-month mark, however, two of the replicates of CPgly survived. At lab A, liquid nitrogen preservation had higher success rates (96.7%) than cryopreservation (33.3%). Conversely, at lab B cryopreservation (50.0%) out-performed liquid nitrogen preservation (23.3%).

#### Neocallimastix

At lab A, LNPc worked best for *Neocallimastix* (93.3% overall success rate). LNPb (40.0% overall survival rate) showed mixed results, with no survivors at the nine-month mark and only one or two replicates surviving at all other time points. None of the other protocols worked for *Neocallimastix* at lab A. At lab B, CPeg and LNPb showed 100% overall survival rates for *Neocallimastix*. Additionally, all three replicates of CPgly were successfully resuscitated after 12 months. Results for CPgly, however, were rather unstable, with no replicates surviving after one week and after six months, but two or more replicates surviving at the three-, nine-, and 12-month mark. At lab A, liquid nitrogen preservation (66.7%) clearly out-performed cryopreservation (0.0%), while at lab B, cryopreservation (73.3%) out-performed liquid nitrogen preservation (56.7%).

### Growth evaluation results

For none of the analyzed combinations between accumulated gas pressure and categorical variables (see below) ANOVA requirements were met. Hence, Kruskal-Wallis including *post hoc* Dunn tests, and Mann-Whitney U tests were performed.

#### Gas pressure measurements of the controls

**FIGURE 5:**
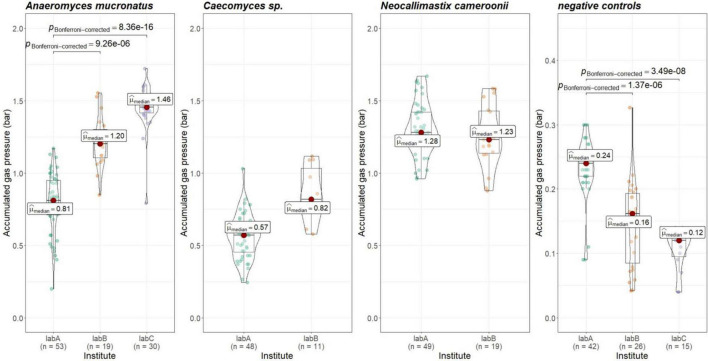
Comparisons of accumulated gas pressure in the positive controls of the three different AF-methanogen co-cultures between the three partnering laboratories (lab A, lab B, lab C). Statistical tests: Kruskal–Wallis with post hoc Dunn-test. Only significant pairwise comparisons are shown. *Caecomyces* sp.: *p* = 3.50e-5, *Neocallimastix cameroonii*: *p* = 0.27.

**FIGURE 6:**
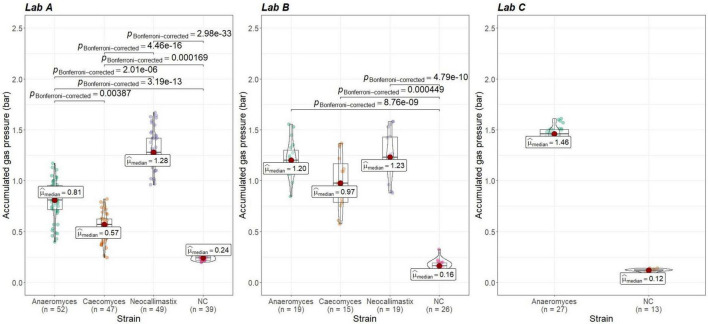
Comparisons of accumulated gas pressure in the positive controls of the three different AF-methanogen co-cultures within the three partnering laboratories (lab A, lab B, lab C). Lab A and Lab B: statistical tests: Kruskal-Wallis with post hoc Dunn-test. Only significant pairwise comparisons are shown. Lab C: statistical test: Mann–Whitney U, *p* = 4.15e-07.

To check whether gas pressure measurements are comparable between institutes, the data from positive and negative controls was evaluated. Gas pressure measurements for *Anaeromyces* were significantly different between all institutes (Kruskal–Wallis, *p* < 0.01, H = 0.498, large effect size). Looking closer, gas pressure measurements for *Anaeromyces* are significantly different between lab A and C, and between lab A and B, but the differences between lab B and C were non-significant (see Figure 5). Accumulated gas pressure in *Caecomyces* positive controls were significantly different between lab A and lab B, but *Neocallimastix* showed no significant difference between lab A and lab B. The negative controls of lab A were significantly different from those of lab B and lab C. At each lab, all strains showed significantly higher gas pressure than the negative controls (see Figure 6). At lab A, however, all strains showed significant differences in gas pressure, while at lab B the differences in gas pressure of the respective positive controls were non-significant.

#### Gas pressure measurements and visual growth evaluation

**FIGURE 7:**
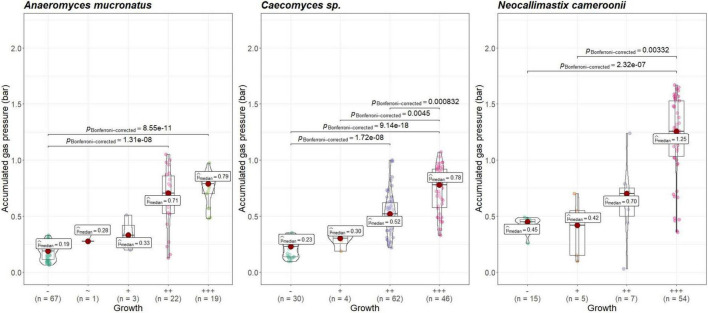
Correlation between AF visual growth inspection and accumulated gas pressure at lab A. Visual growth evaluation categories: “–”, no growth; “+++”, very good growth. Statistical tests: Kruskal–Wallis with *post hoc* Dunn-test. Only significant pairwise comparisons are shown.

**FIGURE 8:**
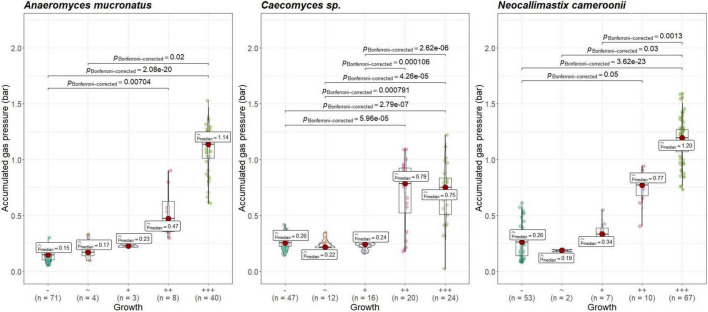
Correlation between AF visual growth inspection and accumulated gas pressure at lab B. *Visual growth evaluation categories: “*–“, no growth; “+++”, very good growth. Statistical tests: Kruskal–Wallis with *post hoc* Dunn-test. Only significant pairwise comparisons are shown.

**FIGURE 9:**
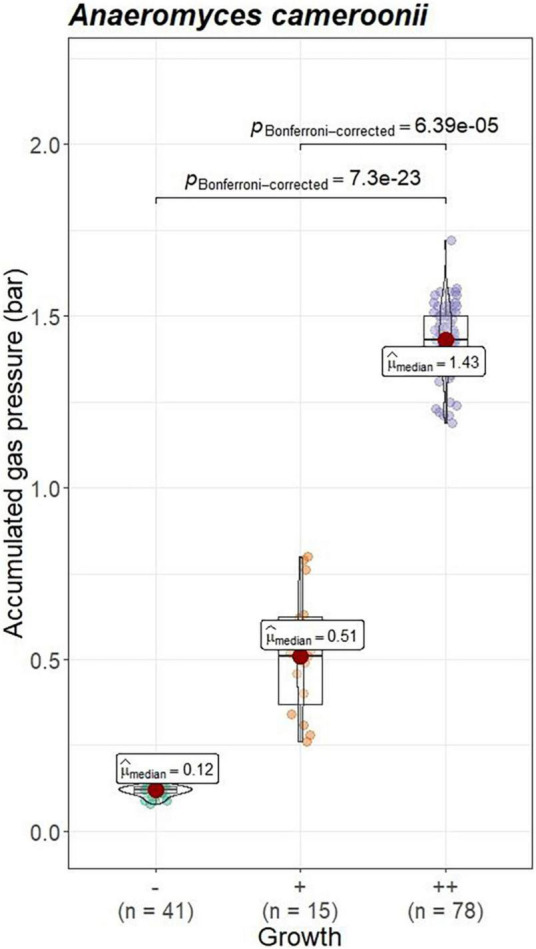
Correlation between AF visual growth inspection and accumulated gas pressure at lab C. Visual growth evaluation categories: “–”, no growth; “++”, very good growth. Statistical tests: Kruskal–Wallis with *post hoc* Dunn-test. Only significant pairwise comparisons are shown.

Visual growth evaluation was hardest for *Caecomyces*, as this culture does not clump the straw and often also does not form distinct biomass flakes. Instead, it mostly forms small biomass flakes (see Supplementary Figure 2C) or grows dispersed throughout the medium looking like white, undissolved powder (see Supplementary Figure 2B). This can hamper visual distinction between (very) good growth and slow, impeded growth. Nevertheless, in all three co-cultures of this study, accumulated gas pressure correlated with subjective visual evaluation of AF growth, i.e., better growing cultures (visually more fungal biomass formed, floating biomass like matts/buoy, etc.) had accumulated more gas pressure (see Figures 7–9). Using visual growth evaluation of AF in conjunction with accumulated gas pressure measurements is essential because it helps differentiating high gas pressure values that were due to methanogenic overgrowth or bacterial contamination from higher pressure values due to better AF growth.

### Bacterial contamination

In total, 6.7% (35 serum bottles out of 525) of resuscitated replicates over all strains, and institutes showed bacterial contamination (verified by turbidity of the medium and microscopy). About 68.6% of these contaminations were found in LNPb bottles (24 out of 35), 11.4% in CPgly bottles (4 out of 35), 8.6% in AP bottles (3 out of 35), 3% in LNPc bottles (3 out of 35), and 2.9% in CPeg bottles (1 out of 35).

## Discussion

The agar preservation (AP) protocol described in this study leaned on a previously described spore extraction protocol (Calkins et al., 2016), that also reported viability of the cultures on agar for up to 4 months. In our set-up, this zoospore extraction was the least successful preservation protocol. AF zoospore production is thought to cease after a few days in batch culture (Mountfort, 1994), and the ability of AF to produce new colonies is likely to decrease with decreasing nutrient content and increasing waste products in the medium. This could explain the low observed success rate of zoospore extraction after months on agar medium without sub-cultivation or feeding. However, if the final goal of this method is not to collect zoospores, the described protocol can be used to preserve mature fungal structures. With trials ran on the same AF overgrown agar we could show that there are two modifications to resuscitate mature fungal structures from agar: (A) Shaking the bottle thoroughly once the medium for extraction was injected (as already described in Calkins et al., 2016) leads to breaking of the agar. This can release mature, viable AF thalli embedded in deeper layers of the agar and suspend them into the liquid. This liquid can then be transferred into a new serum bottle by injection. This method bears least risk of bacterial contamination and led to successful resuscitations for up to 6 months (only tested in *Anaeromyces*). (B) Without adding suspension liquid, the agar bottle can be opened and pieces of agar with mature thalli can be transferred into a new serum bottle with the help of sterile tweezers. This method slightly increases the risk for bacterial contamination but can extend viability for another couple of months (up to twelve months for *Anaeromyces*, and 9 months for *Caecomyces* and *Neocallimastix*). Contrary to previous reports (Solomon et al., 2016), however, handling procedures like this need not be performed in anaerobic conditions (e.g., under constant CO_2_ flow). AF, either encapsulated in pieces of agar or within their biomass balls/matts seem to be rather resistant to short-term oxygen exposure. This experience is corroborated by the reported tolerance to oxygen exposure of different AF strains (Leis et al., 2014). The medium the oxygen-exposed AF biomass is transferred into, however, needs to be anoxic and working in an anoxic environment could potentially further increase success rates, especially for inexperienced handlers. The agar preservation method described in Calkins et al. (2016) and the variants described here could present a reliable, low-cost and low-effort, short term alternative to keeping cultures alive in consecutive batch cultures. Nevertheless, it does not appear to be a dependable method for long-term storage. There, cryopreservation (at –80°C or in liquid nitrogen) might be more reliable.

Cryopreservation of AF was previously done at –80°C or in liquid nitrogen, usually with DMSO or ethylene glycol as cryoprotectants (Yarlett et al., 1986; Sakurada et al., 1995). In recent years, however, glycerol was reported to be more beneficial to *Caecomyces* survival (up to 90 days) at –70°C than ethylene glycol (Nagpal et al., 2012). Contrary to this report, we could not confirm or deny the superiority of glycerol over ethylene glycol in the cryopreservation (–80°C) of *Caecomyces*. At lab A, preservation in glycerol (CPgly) did show a higher success rate than preservation in ethylene glycol stock solution (CPeg) but at lab B, CPeg clearly out-performed CPgly. This indicates that other factors (e.g., handling, biomass, see below) influence resuscitation success more than the preservative used. Glycerol was also reported successful in cryopreservation at –80°C in filamentous AF (Solomon et al., 2016). However, Solomon and collaborators’ results reported for this method were variable between the genera tested (*Anaeromyces*, *Neocallimastix*, and *Piromyces*) and even variable between two strains of the same genus (*Neocallimastix*). In our set-up, ethylene glycol and glycerol were equal in success rates for *Anaeromyces* at lab C. At lab B, the superiority of ethylene glycol over glycerol found in *Caecomyces* was also true for *Anaeromyces* and *Neocallimastix*. At lab A, both protocols, CPeg and CPgly, showed only very limited success for filamentous AF. Still, more research on the efficacy of different preservatives, and the most effective concentrations of those in the preservation medium is needed.

**FIGURE 10:**
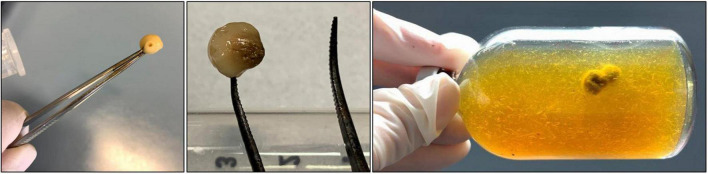
Biomass ball/sphere of an Anaeromyces mucronatus culture grown in a large serum bottle filled with medium A. The diameter of these balls/spheres can be 0.5–10 mm after seven days of growth.

Inconsistency and variability of results is often speculated to be coming from inherent physiological and metabolic differences among different AF genera as well as different species within the same genus. While this is a reasonable assumption considering proven differences in enzymatic activities between species and genera (Paul et al., 2010; Henske et al., 2018), we could show that differences in culture handling between labs also influence results. In our set-up, strains as well as protocols, CRF, and evaluation criteria were shared between labs. Hence, the noticeable differences in the results of the different labs can be attributed to the influence of differences in culture handling. While these differences were noticeable in the results for every AF culture tested in this study, they were most relevant in *Anaeromyces mucronatus*. Polycentric filamentous AF like *Anaeromyces* tend to form big, robust biomass balls with little to no thalli found in the liquid phase of the medium (Figure 10). This phenomenon can be observed both on soluble C-sources (like cellobiose) as well as insoluble ones (like milled wheat straw). Transfer of rhizobium, however, is essential for polycentric cultures since they are known to become sterile over time in batch culture but can grow vegetatively through their polynucleated hyphae (Hanafy et al., 2022). In case of tightly clumped biomass, transfer of biomass can be achieved by opening the serum bottle, taking out the biomass balls with the help of sterile tweezers (see Figure 10), cutting them into pieces, and transferring the pieces into fresh serum bottles containing sterile medium. If done accurately, risk of contamination is low. As described above, oxygen exposure during transfer seems to be a minor issue given that the medium the biomass is transferred into, is anoxic. These additional handling steps are vital for *Anaeromyces* culture survival, as proven by the high survival rates of this strain in lab C. At lab A and lab B, sub-cultivation of this strain was done by injection only, leading to significantly lower success rates.

Differences in culture handling alone, however, do not fully explain variability of results and erratic survival patterns over time within the same institute, strain, and protocol in our set-up. This inconstancy in resuscitation success could indicate that survival depends on other factors than handling or preservation period, like the amount, or the quality of the biomass preserved. Due to the inhomogeneous distribution of AF biomass in liquid medium, transferring a set amount of biomass during sub-cultivation by injection is difficult. This is especially true in filamentous AF that tend to form biomass balls or matts while bulbous AF are more evenly distributed throughout the medium and usually do no form tight biomass clumps. It can, however, also be an issue for bulbous AF on particulate C-sources like milled wheat straw that might clog the cannula used for transfer. Shaking of the serum bottles before sub-cultivation is used to alleviate the clumping problem but has only limited effect due to the stability of biomass clumps. Hence, each new serum bottle is most likely injected with a variable amount of biomass. Besides, this biomass is most likely a mixture of AF in various stages of their life cycle. The life cycle of anaerobic fungi includes motile zoospores that can search for and attach to plant material, encyst there, and grow into mature thalli that again produce zoospores within their sporangia (Gruninger et al., 2014). Depending on the transfer and handling method used, it is probable that freely swimming zoospores or small, immature AF thalli are more easily transferred by injection than mature, clumped biomass. This could lead to subtle differences in growth behavior of each culture and furthermore to variability in results (e.g., visible biomass, gas production) as preservation success could potentially be influenced by the life-cycle stage of AF. Some earlier reports of AF preservation focused on the preservation of zoospores alone (obtained by centrifugation and filtration). This bears the advantage of preserving homogenized and synchronized biomass, but reports showed mixed survival rates for AF after zoospore preservation over one year (40% reported in Yarlett et al., 1986; 80% reported in Sakurada et al., 1995). However, the protocols used in those publications differed greatly and further testing is needed to evaluate the efficacy of zoospore preservation for AF survival.

Some researchers have proposed the existence of another life-cycle stage that can withstand harsh environmental conditions like desiccation or oxygen exposure (Brookman et al., 2000; Hanafy et al., 2022). These ‘resting stages’ could explain reported AF survival in air-dried or frozen feces (Wubah et al., 1991; Davies et al., 1993; McGranaghan et al., 1999) as well as in nutrient deprived medium (Joblin, 1981). It could also explain the extraction of viable AF from months old agar cultures (as seen in this study in protocol AP) if nutrient deprivation and/or changes of environmental conditions triggered resistant body formation. What those ‘resting stages’ could look like or how they are formed remains to be elucidated. Melanization of sporangia and/or zoospores is suspected to play a role since AF thalli in older cultures show dark, brownish pigmentation (Wubah et al., 1991; Brookman et al., 2000). Melanin is also known to be involved in stress tolerance mechanisms of other fungi (Toledo et al., 2017). As of now, however, it is unclear whether the pigmentation in AF is indeed melanin (Brookman et al., 2000). Furthermore, it is currently unclear whether specialized structures are generated for AF survival or if stress is tolerated by adaptations like melanization or thickening of the cell-walls. Elucidating this issue would be highly relevant to refining preservation methods for AF.

In recent years, reports focused primarily on the preservation of mature biomass that contains AF in various life-cycle stages. This is also true for the protocols reported in this study. In protocols CPeg and CPgly, where AF were directly preserved in the serum bottles they were grown in, all the biomass produced during growth was preserved. In protocol LNPc, a fraction of the biomass produced during growth was transferred into cryovials by injection and preserved there. This introduced again the issue of homogenous biomass transfer by injection. In protocol LNPb, concentrated biomass is transferred into cryovials for preservation. Of those three different methods of biomass preservation, LNPb showed the highest success rates, despite some variability between the labs.

Another issue related to handling procedures is bacterial contamination. Especially if bottles are opened, introduction and proliferation of bacteria might occur despite the addition of antibiotics. In the present study, contamination was observed mainly in protocol LNPb. While this proves that manual handling (i.e., transfer into cryovials, opening of serum bottles, transfer with tweezers, etc.) introduces a higher risk of bacterial contamination, it also shows that if care is taken, the risk can be minimal (35 out of 525 resuscitation bottles were contaminated).

To summarize the findings of this study, the transfer of AF biomass clumps into cryovials together with ethylene glycol stock solution and their preservation in liquid nitrogen (protocol LNPb) showed highest success rates in general, followed by directly preserving AF in serum bottles in ethylene glycol stock solution at –80°C (protocol CPeg). Variability due to differences in handling can have a large impact on results from different labs. Regardless of the protocol used, it is advisable to preserve AF cultures (either pure culture or in co-culture with their natural syntrophs) in several replicates immediately after completion of the isolation and identification process. For short-term storage, isolated AF cultures should be immediately preserved on agar in several replicates. Additionally, several replicates should be either stored in liquid nitrogen (following the LNPb protocol) or at –80°C (following the CPeg protocol). This measure not only serves as a culture back-up but also limits genetic drift of the AF isolate (and its syntrophic partners) that could happen if they were kept in consecutive batch cultures over longer periods of time. Whether anaerobic fungal cultures preserved by the methods presented in this study could be viable for more than 12 months remains to be elucidated.

## Data availability statement

The original contributions presented in this study are included in the article/Supplementary material, further inquiries can be directed to the corresponding author.

## Author contributions

JV performed the experiments at one lab, did the data analysis, wrote the manuscript, provided pictures and gave consent for their publication, created the figures, helped shape the experimental design, and has first authorship. AJ performed the experiments at one lab and created the experimental design. DY performed the experiments at one lab, helped shape the experimental design and the manuscript, and provided pictures of *Anaeromyces* cultures and gave consent for their publication. LB, NP, LM, KL, and MN contributed equally to this work by helping with the experiments at the participating labs. HI helped shape the experimental design. VF helped shape the experimental design and the manuscript. SMP helped shape the experimental design and this manuscript and has senior authorship. All authors contributed to the article and approved the submitted version.

## Abbreviations

AP: agar preservation
CPeg: cryopreservation (–80 ° C) of liquid culture in ethylene glycol stock solution
CPgly: cryopreservation (–80 ° C) of liquid culture in glycerol stock solution
LNPb: liquid nitrogen preservation of concentrated biomass in ethylene glycol stock solution
LNPc: liquid nitrogen preservation of liquid culture in ethylene glycol stock solution.
