# Supplementary material for: No time to die: Comparative study on preservation protocols for anaerobic fungi
Source: Front Microbiol. 2022 Sep 26;13:978028. doi: 10.3389/fmicb.2022.978028 (PMC9549207; doi:10.3389/fmicb.2022.978028)

Supplementary Material

No Time to Die: Comparative Study on Preservation Protocols for Anaerobic Fungi

Julia Vinzelj^1*^, Akshay Joshi^1,2^, Diana Young^3^, Ljubica Begovic^1^, Nico Peer^1^, Lona Mosberger^2^, Katharina Cécile Schmid Luedi^2^, Heribert Insam^1^, Veronika Flad^3^, Magdalena Nagler^1^, Sabine Marie Podmirseg^1^

^1^Department of Microbiology, University of Innsbruck, Innsbruck, Austria

^2^Institute of Chemistry and Biotechnology, Biocatalysis and Process Technology Unit, Zurich University of Applied Science, Waedenswil, Switzerland

^3^Micro- and Molecular Biology, Central Department for Quality Assurance and Analytics, Bavarian State Research Center for Agriculture, Freising, Germany

*** Correspondence:**Julia Vinzelj
[julia.vinzelj@uibk.ac.at](mailto:julia.vinzelj@uibk.ac.at)

Keywords: Neocallimastigomycota_1_, cryopreservation_2_, anaerobic fungi_3_, long-term storage_4_, short-term storage_5_, preservation techniques_6_, resting stages_7_, culture preservation_8_

# Supplementary Table

## Supplementary table 1: Count of successful resuscitation at each lab for each timepoint, protocol, and strain. Per lab, strain, timepoint, and protocol, three resuscitations were attempted. *Anaeromyces* = *Anaeromyces mucronatus*, *Caecomyces* = *Caecomyces sp.*, *Neocallimastix* = *Neocallimastix cameroonii*. AP = Agar preservation protocol, CPeg = cryopreservation protocol with ethylene glycol stock solution, CPgly = cryopreservation protocol with glycerol stock solution, LNPb = preservation of liquid culture with ethylene glycol stock solution in liquid nitrogen, LNPc = preservation of liquid culture with ethylene glycol stock solution in liquid nitrogen


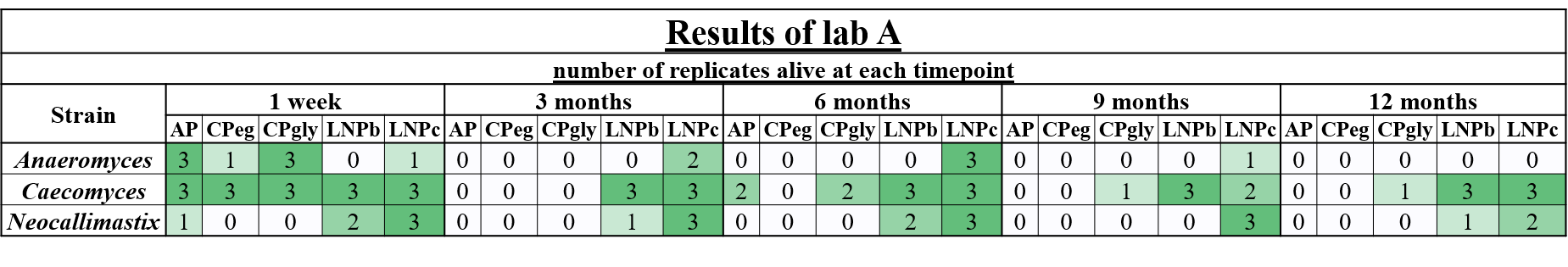

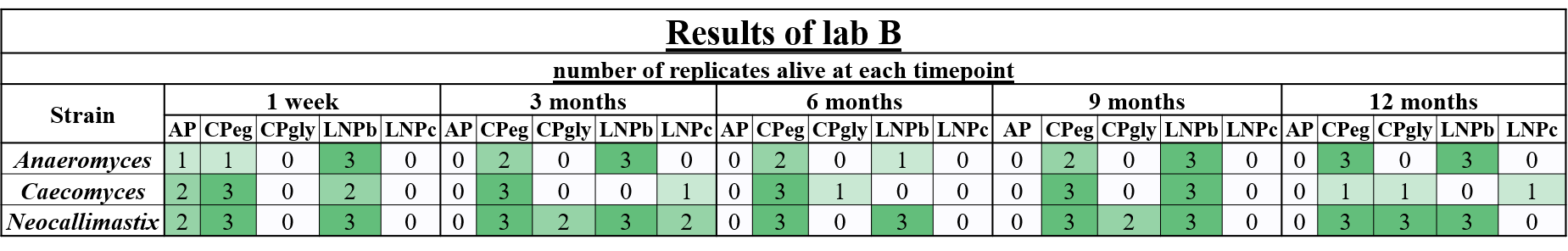

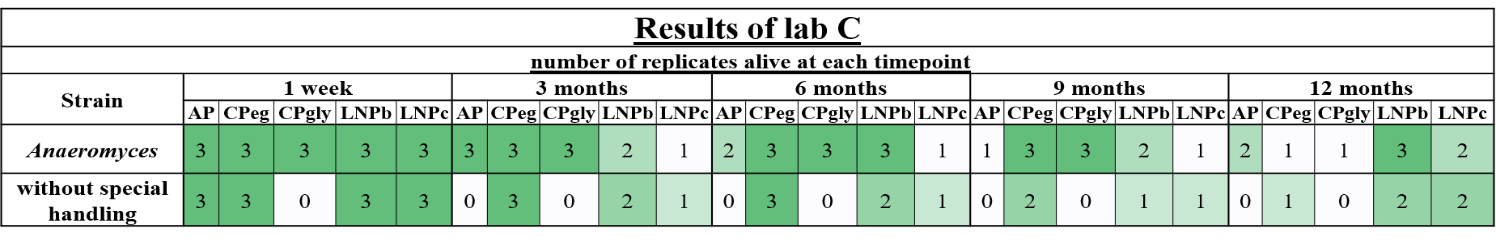

Supplement: Supplementary file 1 [file Table_1.docx]
