# Supplementary material for: No time to die: Comparative study on preservation protocols for anaerobic fungi
Source: Front Microbiol. 2022 Sep 26;13:978028. doi: 10.3389/fmicb.2022.978028 (PMC9549207; doi:10.3389/fmicb.2022.978028)
Supplement: Supplementary file 3 [file Presentation_1.pdf]

No Time to Die: Comparative Study on Preservation Protocols for Anaerobic Fungi

Supplementary Material

Julia Vinzelj[1*], Akshay Joshi[1,2], Diana Young[3], Ljubica Begovic[1], Nico Peer[1], Lona Mosberger[2], Katharina Cécile Schmid Luedi[2], Heribert Insam[1], Veronika Flad[3], Magdalena Nagler[1], Sabine Marie Podmirseg[1]

[1]Department of Microbiology, University of Innsbruck, Innsbruck, Austria

[2]Institute of Chemistry and Biotechnology, Biocatalysis and Process Technology Unit, Zurich University of Applied Science, Waedenswil, Switzerland

[3]Micro- and Molecular Biology, Central Department for Quality Assurance and Analytics, Bavarian State Research Center for Agriculture, Freising, Germany

*** Correspondence:**
Julia Vinzelj
julia.vinzelj@uibk.ac.at

Supplementary table 1: Count of successful resuscitation at each lab for each timepoint, protocol, and strain. Per lab, strain, timepoint, and protocol, three resuscitations were attempted. *Anaeromyces* = *Anaeromyces mucronatus*, *Caecomyces* = *Caecomyces sp.*, *Neocallimastix* = *Neocallimastix cameroonii*. AP = Agar preservation protocol, CPeg = cryopreservation protocol with ethylene glycol stock solution, CPgly = cryopreservation protocol with glycerol stock solution, LNPb = preservation of liquid culture with ethylene glycol stock solution in liquid nitrogen, LNPc = preservation of liquid culture with ethylene glycol stock solution in liquid nitrogen

Results of lab A

number of replicates alive at each timepoint

Strain	1 week					3 months					6 months					9 months					12 months				
	AP	CPeg	CPgly	LNPb	LNPc	AP	CPeg	CPgly	LNPb	LNPc	AP	CPeg	CPgly	LNPb	LNPc	AP	CPeg	CPgly	LNPb	LNPc	AP	CPeg	CPgly	LNPb	LNPc
Anaeromyces	3	1	3	0	1	0	0	0	0	2	0	0	0	0	3	0	0	0	0	1	0	0	0	0	0
Caecomyces	3	3	3	3	3	0	0	0	3	3	2	0	2	3	3	0	0	1	3	2	0	0	1	3	3
Neocallimastix	1	0	0	2	3	0	0	0	1	3	0	0	0	2	3	0	0	0	0	3	0	0	0	1	2

Results of lab B

number of replicates alive at each timepoint

Strain	1 week					3 months					6 months					9 months					12 months				
	AP	CPeg	CPgly	LNPb	LNPc	AP	CPeg	CPgly	LNPb	LNPc	AP	CPeg	CPgly	LNPb	LNPc	AP	CPeg	CPgly	LNPb	LNPc	AP	CPeg	CPgly	LNPb	LNPc
Anaeromyces	1	1	0	3	0	0	2	0	3	0	0	2	0	1	0	0	2	0	3	0	0	3	0	3	0
Caecomyces	2	3	0	2	0	0	3	0	0	1	0	3	1	0	0	0	3	0	3	0	0	1	1	0	1
Neocallimastix	2	3	0	3	0	0	3	2	3	2	0	3	0	3	0	0	3	2	3	0	0	3	3	3	0

Results of lab C

number of replicates alive at each timepoint

Strain	1 week					3 months					6 months					9 months					12 months				
	AP	CPeg	CPgly	LNPb	LNPc	AP	CPeg	CPgly	LNPb	LNPc	AP	CPeg	CPgly	LNPb	LNPc	AP	CPeg	CPgly	LNPb	LNPc	AP	CPeg	CPgly	LNPb	LNPc
Anaeromyces	3	3	3	3	3	3	3	3	2	1	2	3	3	3	1	1	3	3	2	1	2	1	1	3	2
**without special handling**	3	3	0	3	3	0	3	0	2	1	0	3	0	2	1	0	2	0	1	1	0	1	0	2	2

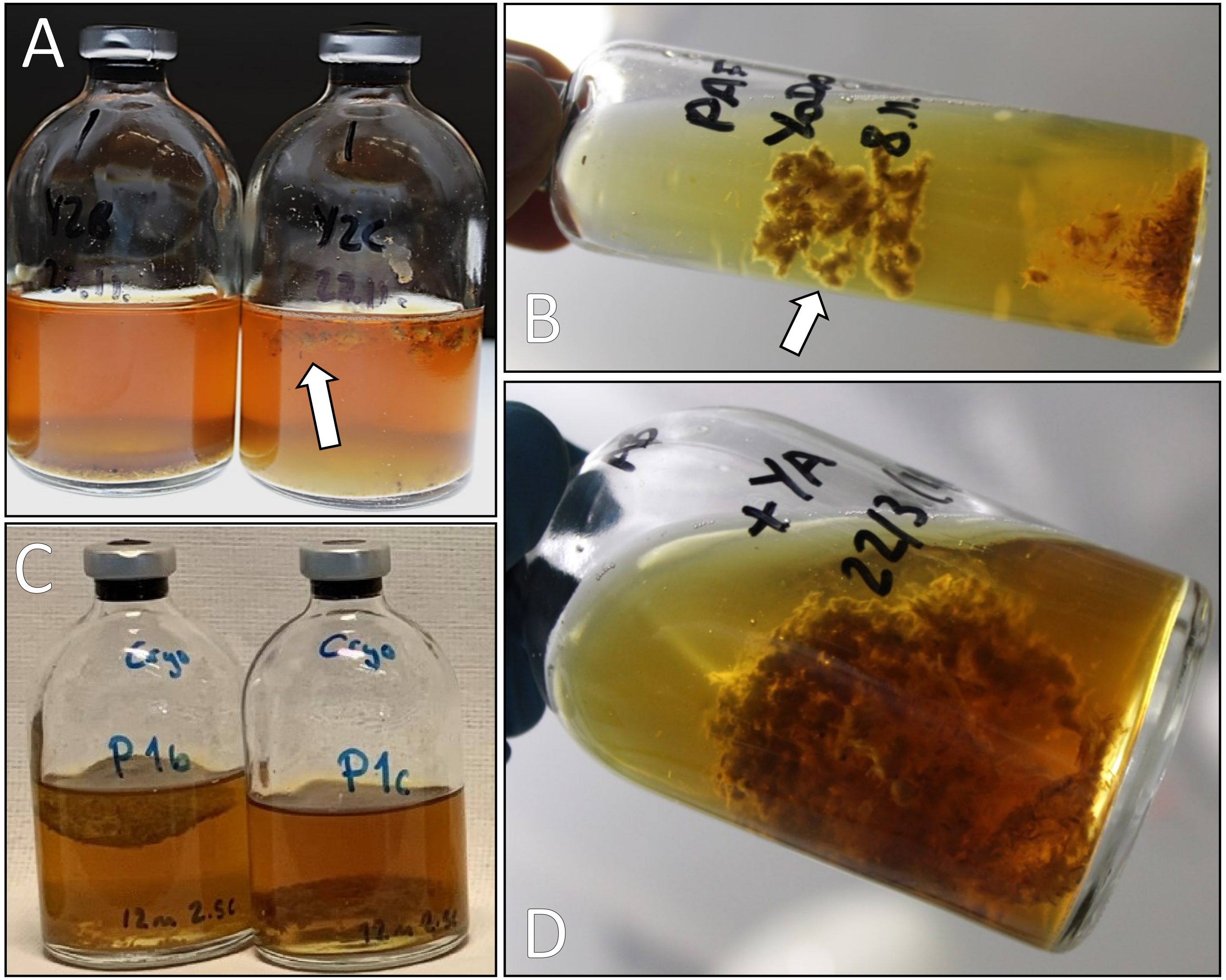

Supplementary figure 1: Growth of *Anaeromyces mucronatus*, visual inspection. **(A)** no growth (left) and a little growth (right), **(B)** good growth (biomass balls/spheres), **(C)** very good growth (biomass matt/buoy) **(D)** very good growth (biomass matt as well as balls/spheres). The arrows indicate AGF biomass balls/spheres.

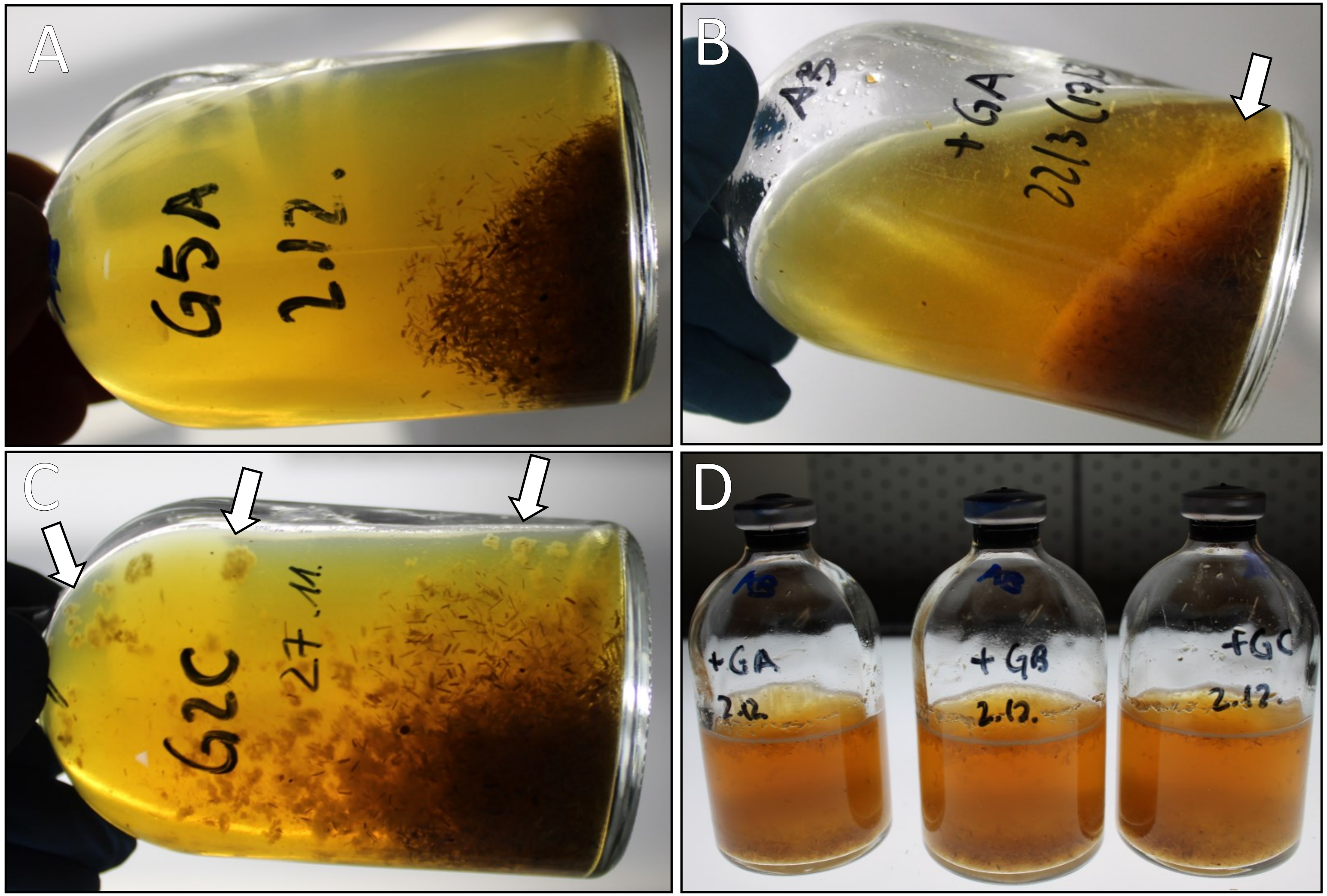

Supplementary figure 2: Growth of *Caecomyces* sp., visual inspection. **(A)** no growth, **(B)** a good growth (small biomass flakes), **(C)** and **(D)** very good growth (bigger biomass flakes). The arrows indicate AGF biomass flakes.

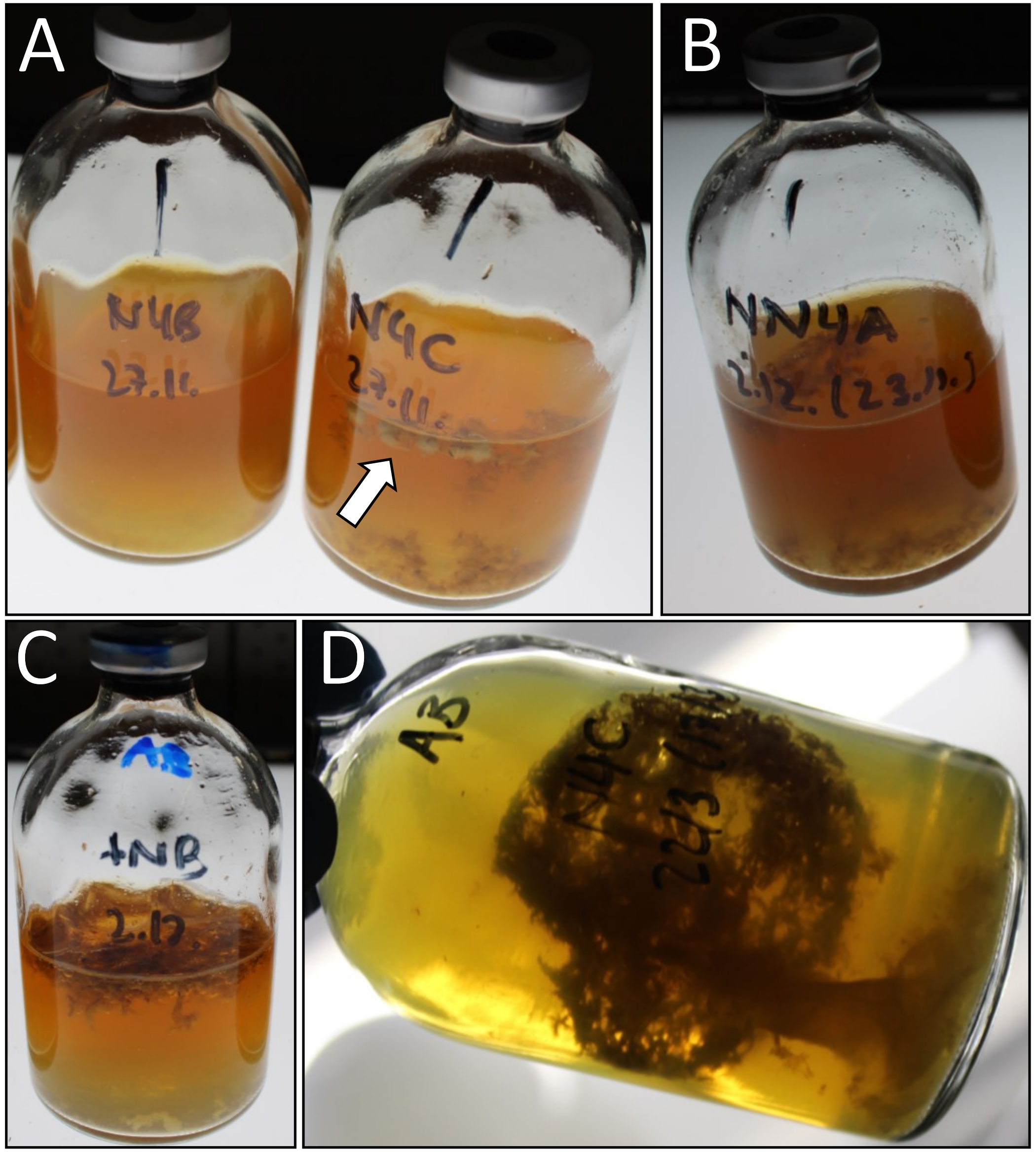

Supplementary figure 3: Growth of *Neocallimastix cameroonii*, visual inspection. **(A)** no growth (left) and a little growth (biomass balls), **(B)** a little growth (biomass matts/clumps), **(C)** and **(D)** very good growth (biomass matt/buoy). The arrows indicate AGF biomass balls/spheres.

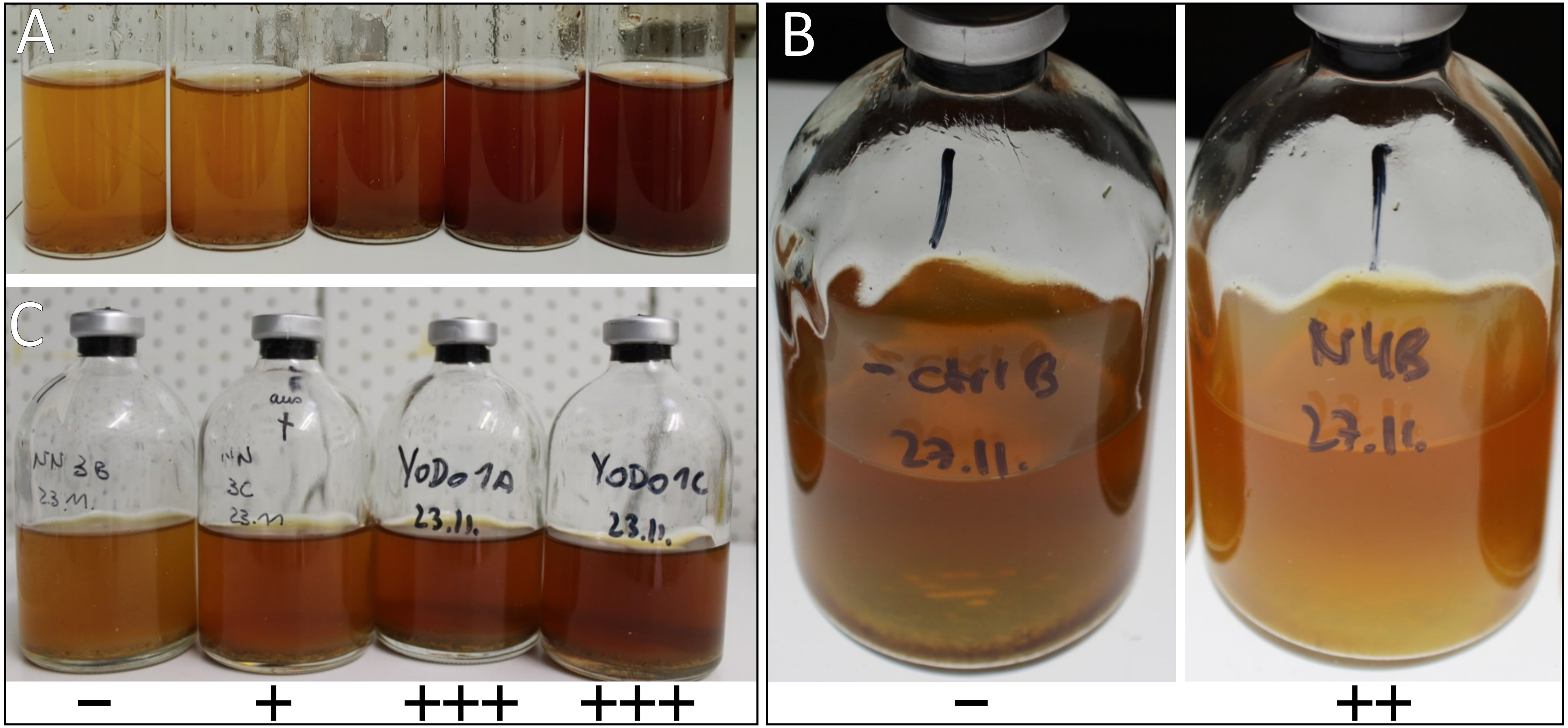

Supplementary figure 4: (A) (C) Visual evaluation of the medium color, ranging from “-” (not red) to “+++” (very red). Since resazurin is used as redox indicator in this medium, the red color indicates less anoxic conditions. **(B)** Visual evaluation of medium turbidity, ranging from “-” (not turbid) to “++” (very turbid).
